# Supplementary material for: Rapid turnover of life-cycle-related genes in the brown algae
Source: Genome Biol. 2019 Feb 14;20:35. doi: 10.1186/s13059-019-1630-6 (PMC6374913; doi:10.1186/s13059-019-1630-6)
Supplement: Supplementary file 3 — Protein alignments, corresponding Gblocks html file and CDS sequences for the lineage conserved gametophyte-biased genes. (ZIP 2109 kb) [file 13059_2019_1630_MOESM3_ESM.zip › Con_GA_alignments/Ec-26_003400.aa_ali.fasta-gb.htm]

Ec-26\_003400.aa.fasta.trans.aa\_ali.fasta


## Gblocks 0.91b Results

Processed file: **Ec-26\_003400.aa.fasta.trans.aa\_ali.fasta**  
Number of sequences: **2**  
Alignment assumed to be: **Protein**  
New number of positions: **284** (selected positions are underlined in blue)

```
                         10        20        30        40        50        60
                 =========+=========+=========+=========+=========+=========+
Ec-26_003400     ------------------------MALVSTETQLRSMSAGPERFMTMNLMSSGPCTVSKK
MSTRG.29301.1    XQSVVRKRRSVGTCPESQKSLSAKMALVSTEMQLRSMSPGPERFMTMNLMSSGPCTVGNG
                                         ####################################


                         70        80        90       100       110       120
                 =========+=========+=========+=========+=========+=========+
Ec-26_003400     ATSYGRTVRRLNTRDNHTTADVDGAQTFVRTSTLTNKPDSYGVADILGTGSMRLHKDTNK
MSTRG.29301.1    VTSYGRTVRRMHTRDNHTTADVDGARTFVRTSTLTNKPDSHGVADIRGTGSIRLHKDTNK
                 ############################################################


                        130       140       150       160       170       180
                 =========+=========+=========+=========+=========+=========+
Ec-26_003400     PDRQLYHDDIEGSNPRNKWLFRTTRCIDPLEPEYTLPSFTTAPPIAPKFTRDSHDVSDIE
MSTRG.29301.1    PDRQLYHDDIEGSNPRNKWLFRTRRCVNPLEPEYTLPSFVTAPAVAAKFTRDSYDVSDID
                 ############################################################


                        190       200       210       220       230       240
                 =========+=========+=========+=========+=========+=========+
Ec-26_003400     GTKSRPLYPLEQRHNTLVDDIEGAQSGWKPRHRRARHEAAPLDHCLNVSDITGGGFRTRR
MSTRG.29301.1    GTKSRPLYPLEQRESHLVDDIEGAKSGWKPRHRRARHEAAPLDHSLNVSDITAGGFRTRR
                 ############################################################


                        250       260       270       280       290       300
                 =========+=========+=========+=========+=========+=========+
Ec-26_003400     ATNPLTPSYRVNGMDVADDPVKSRPRALPKAKDGPFYPLTTADIEGATPGWRPLPQGRGR
MSTRG.29301.1    ATNPLTPSYHVNGMDVADDPVKSRPRPMPRAKDGPFYPLTTADIEGATPGWRPLPQ----
                 ########################################################    


                        310       320       330       340       350       360
                 =========+=========+=========+=========+=========+=========+
Ec-26_003400     QLARVAPTRPTRFEDNKSAVVRRDPGRWSPRGQLQHQQQERDKRADPATRERCSAVTERQ
MSTRG.29301.1    ----VNPPLEARRHFRNTNFMGDIPG--AQADTVKHAICTK-RHVNPLDPVYHSLDGEP-
                                                               ############  


                        370       380
                 =========+=========+========
Ec-26_003400     RGLAADQNSSDTWNRDQKQQQPRRRQQK
MSTRG.29301.1    --LANPQTPLYKEPACVEAEAQLDRSIA
```

```
Parameters used
Minimum Number Of Sequences For A Conserved Position: 2
Minimum Number Of Sequences For A Flanking Position: 2
Maximum Number Of Contiguous Nonconserved Positions: 8
Minimum Length Of A Block: 10
Allowed Gap Positions: None
Use Similarity Matrices: Yes
```

```
Flank positions of the 2 selected block(s)
Flanks: [25  296]  [347  358]  

New number of positions in Ec-26_003400.aa.fasta.trans.aa_ali.fasta-gb:  284  (73% of the original 388 positions)
```
